# The PTENP1 Pseudogene, Unlike the PTEN Gene, Is Methylated in Normal Endometrium, As Well As in Endometrial Hyperplasias and Carcinomas in Middle-Aged and Elderly Females

**Published:** 2018

**Authors:** T. F. Kovalenko, K. V. Morozova, L. A. Ozolinya, I. A. Lapina, L. I. Patrushev

**Affiliations:** Shemyakin-Ovchinnikov Institute of Bioorganic Chemistry Russian Academy of Sciences, Miklukho- Maklaya Str. 16/10, Moscow, 117997, Russia; Pirogov Russian National Research Medical University, Ostrovitjanova Str. 1, Moscow, 117997, Russia

**Keywords:** endometrial carcinoma, endometrial hyperplasia, DNA methylation, PTEN, PTENP1, long non-coding RNA

## Abstract

The tumor suppressor PTEN controls multiple cellular functions, including cell cycle, apoptosis, senescence, transcription, and mRNA translation of numerous genes. In tumor cells, PTEN is frequently inactivated by genetic mutations and epimutations. The aim of this study was to investigate the methylation patterns of the *PTEN *gene and its pseudogene *PTENP1 *as potential genetic markers of endometrial hyperplasia (EH) and endometrial carcinoma (EC). Methylation of the 5’-terminal regions of the *PTEN *and *PTENP1 *sequences was studied using methyl-sensitive PCR of genomic DNA isolated from 57 cancer, 43 endometrial hyperplasia, and normal tissue samples of 24 females aged 17–34 years and 19 females aged 45–65 years, as well as 20 peripheral venous blood samples of EC patients. None of the analyzed DNA samples carried a methylated *PTEN *gene. On the contrary, the *PTENP1 *pseudogene was methylated in all analyzed tissues, except for the peripheral blood. Comparison of *PTENP1 *methylation rates revealed no differences between the EC and EH groups (0.80 < *p* < 0.50). In all these groups, the methylation level was high (71–77% in patients *vs*. 58% in controls). Differences in *PTENP1* methylation rates between normal endometrium in young (4%) and middle-aged and elderly (58%) females were significant (*p* < 0.001). These findings suggest that *PTENP1 *pseudogene methylation may reflect age-related changes in the body and is not directly related to the endometrium pathology under study. It is assumed that, depending on the influence of a methylated *PTENP1 *pseudogene on *PTEN *gene expression, the pseudogene methylation may protect against the development of EC and/or serve as a marker of a precancerous condition of endometrial cells.

## INTRODUCTION

Endometrial carcinoma (EC) is one of the most common cancers of the female reproductive tract, with the EC rate accounting for 4.8% of all cancers in females [1]. The risk of EC increases with age: by the age of 75, the cumulative risks of the disease reach 1%, and deaths – 0.2%. Although EC is considered as a postmenopausal disease that develops in females older than 50 years, up to 14% of clinical EC cases are diagnosed in premenopausal age; of these, only 5% occurr in females under 40 years [2-4]. Growing rates of obesity and metabolic syndrome in the populations of Europe and North America, which are accompanied by an increase in the level of endogenous estrogens, as well as the general aging of the population in these countries suggest a significant increase in the incidence rate of the disease in these regions in the near future [5]. All these facts dictate the need to study etiology, as well as search for biomarkers, for an early diagnosis of EC to prevent and provide for timely adequate treatment of the disease.

Depending on the field of application in medicine, biomarkers are usually divided into prognostic, predictive, and pharmacodynamic [6]. Biomarkers of the first type are used to assess a disease’s severity and the survival rate of patients regardless of the treatment. Predictive biomarkers predict the response of patients to the treatment, and pharmacodynamic biomarkers predict the patient’s response to drugs, with allowance for the genetic characteristics of the molecular targets of the used drugs, as well as the enzymes of their metabolism.

On the basis of biomarkers, ECs are traditionally divided into two subtypes [1, 7-9]. The most common and usually sporadic type I Es are usually characterized by the presence of highly differentiated cells and are histologically endometrioid, with tumor cells having the normal diploid karyotype and microsatellite instability (MSI) and expressing estrogen receptors (ERs) and progesterone receptors (PRs). In the case of type I ECs, mutations in the *TP53 *tumor suppressor gene are rare and patients have a good chance of recovering. In contrast, type II ECs do not belong to endometrioid tumors and contain low-differentiated cells, many of which are characterized by aneuploidy, an absence of genetic changes in the p53 protein, and lack of ER and PR expression. In this case, the disease course has an unfavorable prognosis. Based on the data of a histological and molecular genetic analysis, type II ECs may be divided into several additional subtypes, including serous and clear-cell ECs, as well as sarcinosarcoma [1]. A recent meta-analysis of mutations in endometrial tumors using deep sequencing of genomic DNA also revealed a significant heterogeneity of their mutational spectra and enabled researchers to divide ECs into four groups [7].

One of the risk factors for EC is the hyperplastic processes in the endometrium, which occur in a setting of an imbalance of endogenous steroid hormones: estrogens and progesterone [10-12]. EH is characterized by excessive proliferation of cells, which is accompanied by typical morphological changes in tissue. According to the 1994 WHO classification, endometrial hyperplasia includes EH without atypia and EH with atypia, which, in turn, are divided into simple and complex forms [10, 11]. Timely identified EHs usually respond well to therapy. However, complex EHs without atypia and with atypia are transformed into EC in approximately 25 and 50% of cases, respectively [13]. Both cancer and hyperplasia are associated, to varying degrees, with control over cell proliferation, which is accompanied by an increase in the number of cells per unit volume of tissue.

Mutations in the genes of the PI3K/AKT signaling pathway are more typical of EC cells than other types of tumor cells [7, 14]. Serine-threonine protein kinase AKT regulates many cellular functions [15]. The most important negative regulator of signal transduction through this pathway is dual specificity phosphatase PTEN. Mutations in the *PTEN *gene that is located on chromosome 10q23.3 are often detected in EH and in 93% of EC cases. The main PTEN substrate is a secondary messenger phosphatidylinositol-(3,4,5)-trisphosphate (PIP3); under the action of PTEN, PIP3 loses the 3’-phosphate group, transforming into PIP2. Dephosphorylated PIP2 is incapable of activating AKT, which blocks signal transduction through this pathway and suppresses many cellular activities and functions, including cell cycle, apoptosis, cell mobility and polarity, cellular senescence, stem cell renewal, and transcription and translation processes. The suppressor properties of PTEN are also associated with its alternative protein phosphatase activity that is involved in the dephosphorylation of pro-apoptotic proteins, protein kinases, and transcription factors [15-17]. Recently, extracellular and intranuclear suppressor PTEN functions independent of its phosphatase activity were discovered [18]. All these facts suggest that PTEN is an important prognostic and predictive biomarker of carcinogenesis [19] and shed light on the molecular mechanisms of PTEN involvement in the etiology of EC and emphasize the need to study the regulation of its activity in health and in EC.

In normal tissues, the *PTEN *gene is expressed constitutively and its functions are under strict control [17, 20]. The *PTEN *activity is regulated at all levels of its expression: via activation and suppression of transcription [21-23]; post-transcriptionally, at the mRNA level, with the involvement of numerous microRNAs [24, 25]; at the post-translational level, through covalent modifications to a protein product and interactions with numerous membrane, cytoplasmic, and nuclear proteins [20]. Both positive and negative control over the *PTEN* gene transcription involves several transcription factors, and suppression of *PTEN *transcription involves epigenetic mechanisms. The latter include methylation of the promoter DNA regions of the *PTEN *gene, as well as chromatin histone deacetylation at the promoter. Therefore, the available data demonstrate that inactivation of the *PTEN *tumor suppressor gene, which is associated with tumor progression in EH and EC, can occur under the influence of both genetic and epigenetic mutations.

The processed (intron-free) pseudogene *PTENP1* located on chromosome 9p13.3 has 98.6% homology with the functional *PTEN *gene but does not express the PTEN protein, due to mutation-induced loss of the translation initiation codon [26]. *PTENP1 *is usually transcribed to form three long non-coding RNAs (lncRNAs): one sense RNA (sRNA) and two antisense, α and β, RNAs (asRNAs) [27]. Transcription occurs from two opposite overlapping promoters, and the resulting transcripts perform important regulatory functions: sRNA exhibits competing endogenous RNA (ceRNA) properties in the cell [28-30]. According to this mechanism, the microRNA (miRNA) binding sites, MREs, located on pseudogene sRNAs compete with *PTEN* gene mRNA MREs for the specific miRNAs interacting with them, preventing their inhibitory effect on the translocation of PTEN mRNA. Polyadenylated asRNA-β acts similarly. It interacts with the 5’-end of non-polyadenylated PTENP1-sRNA and stabilizes it, enabling a higher competing action of the latter. In contrast, asNRNA-α enables delivery of at least two proteins involved in chromatin rearrangement (DNA methyltransferase 3A (DNMT3A) and the enhancer of Zeste homolog 2 (EZH2)) to the *PTEN *gene promoter [27]. These proteins enable histone H3 lysine 27 trimethylation (H3K27me3), a marker of inactive chromatin with suppressed transcription. A divergent effect of *PTENP1 *pseudogene transcripts on the expression of the *PTEN *tumor suppressor gene suggests the need for fine regulation of their ratio in the cell. The mechanisms of this regulation, which may be impaired in tumors, have not been studied. Indeed,* PTENP1 *pseudogene deletions have been found in sporadic rectal tumors [28], as well as in primary and metastatic melanoma [31]. Another potential mechanism of* PTENP1 *pseudogene inactivation by methylation of its promoter region was identified in lung cancer [32] and, recently, in clear cell renal cancer [33].

Previously, we had discovered methylation of the 5’-terminal promoter region of the *PTENP1 *pseudogene in EC and EH [34]. In the present work, *PTENP1* methylation in EC and EH was studied in detail and methylation of the studied pseudogene region was, for the first time, detected in normal endometrial cells of middle-aged and elderly (MAE) females. In addition, we analyzed the methylation status of *PTEN *gene promoter regions that had not been previously studied in EC and EH.

## EXPERIMENTAL

**Patients and tissue samples**

In this study, we used tissue samples from 143 female patients from the Blokhin Russian Cancer Research Center and Moscow Clinical Hospitals No. 4 and 55. The study included tissue samples from 57 EC patients (mean age, 61.9 ± 7.8 years) and 43 patients with simple endometrial hyperplasia (mean age, 52.1 ± 6.5 years). In addition, we studied peripheral venous blood in 20 EC patients from the main group (mean age, 57.1 ± 7.6 years) who were detected with *PTENP1 *methylation in the endometrium. The control group consisted of 43 females with a histologically intact endometrium who were examined because of suspicion of endometrial precancer. This group included two subgroups: 24 females aged 17 to 34 years (mean age, 24.2 ± 4.8 years) and 19 females aged 45 to 65 years (mean age, 52.5 ± 6.0 years). Comparison of the mean age in the control subgroup (45–65 years) and the group of EC patients (57 patients) using the Mann-Whitney test revealed statistically significant differences (*p * < 0.05). For this reason, when comparing *PTENP1 *pseudogene methylation, we excluded females older than 59 years from the group of EC patients and allocated a subgroup of 24 patients (48–59 years; mean age, 54.3 ± 3.4 years) who did not differ from the controls in this parameter (*p* = 0.095 ). We used both fresh-frozen tissues obtained during surgery or biopsy and samples fixed with formalin and embedded in paraffin blocks. Peripheral venous blood was collected by puncturing the ulnar vein. A 3.8% sodium citrate solution was added at a 1 : 9 ratio as an anticoagulant. The EC stages were classified in accordance with the International Federation of Obstetrics and Gynecology (FIGO) recommendations. The histological EC type was identified in accordance with the World Health Organization recommendations. Consent to data processing was obtained from all patients included in the study.

**Isolation and bisulfite conversion of DNA**

Genomic DNA was isolated by a standard technique using phenol and guanidine chloride [35]. In the case of tissue samples embedded in paraffin blocks, each block was ground to 10 μm fragments using a microtome. Next, a sample was deparaffinized and DNA was extracted using a slightly modified procedure [36]. The DNA concentration was determined using a Qubit fluorometer (Invitrogen, USA). The bisulfite conversion was performed using an EpiTect Bisulfite Kit (Qiagen, Germany) according to the manufacturer’s protocol.

**Methyl-sensitive PCR**

**Fig. 1:**
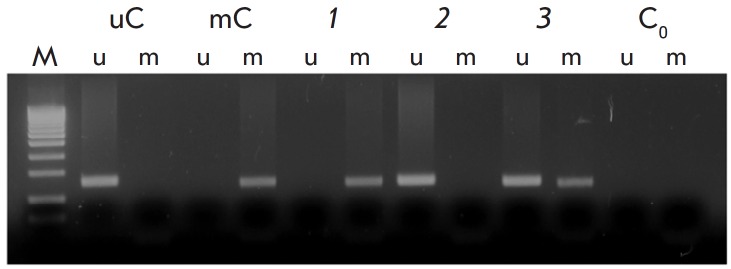
Analysis of the methylation status of the *PTENP1* pseudogene 5’-terminal region by methyl-sensitive PCR. U and m are products of PCR amplification with primers to an unmethylated or methylated template, respectively. Bisulfite-treated DNA templates: uC – a peripheral blood sample (unmethylated control); mC – a peripheral blood sample methylated by SssI methylase (methylated control); 1–3 – tumor DNA samples with (1) methylated* PTENP1*, (2) unmethylated *PTENP1*, and (3) partially methylated *PTENP1 *(some of the tumor cells had unmethylated* PTENP1*); C0 – amplification without a DNA template; M – a molecular weight marker

**Fig. 2:**
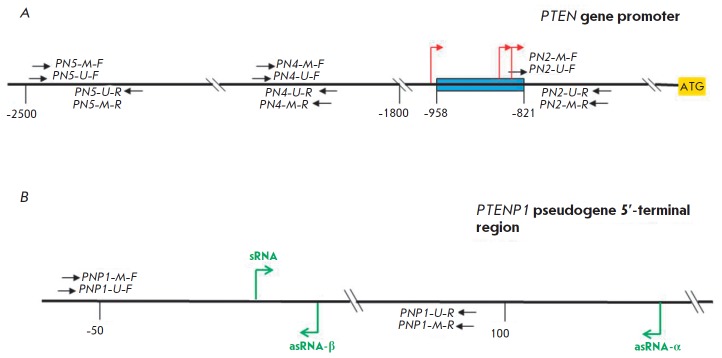
Studied regions of the *PTEN *gene (A) and *PTENP1 *pseudogene (B). A blue rectangle denotes the minimum promoter region of the *PTEN *gene; bent red and green arrows denote the main transcription initiation sites of the* PTEN *gene and of *PTENP1 *pseudogene, respectively; black arrows indicate the location of primers for MS PCR; sRNA is sense *PTENP1 *RNA; asRNA-α(-β) is antisense *PTENP1 *RNA-α(-β); the numbers denote the distance from the ATG codon (A) or sRNA synthesis start site (B)

The reaction mixture (25 μL) contained 67 mM Tris- HCl (pH 8.8), 16.6 mM (NH_4_)_2_SO_4_, 0.01% Tween 20, 2 mM MgCl_2_, four deoxyribonucleoside triphosphates (0.2 mM each; Sibenzyme, Russia), forward and reverse primers (0.5 μM each), 25 ng of bisulfite converted DNA, and 0.5–1.0 units of Taq-DNA polymerase. Taq-DNA polymerase was produced using the recombinant *E. coli* PVG-A1 strain according to a slightly modified procedure by Patrushev et al. [37]. As a fully methylated control, we used DNA isolated from human blood lymphocytes, methylated with SssI methylase (Sibenzyme, Russia) *in vitro*, and treated with sodium bisulfite. Sodium bisulfite-converted DNA from blood lymphocytes served as unmethylated control. After PCR on a Mastercycler pro thermal cycler (Eppendorf, Germany), amplification products were analyzed by electrophoresis on a 3% agarose gel stained with ethidium bromide. A 100 + 50 bp DNA marker (Sibenzyme, Russia) was used as a molecular weight marker. A typical result obtained using methyl-sensitive PCR is shown in *Fig. 1*. Primer sequences, as well as PCR amplification conditions for each pair of primers, are presented in *Table 1*. Annealing temperature for each pair was optimized in a temperature gradient to exclude nonspecific annealing of primers. In addition, the optimal number of cycles was determined to prevent the formation of nonspecific PCR products due to over-amplification. The primers reported in [32] were used for the analysis of the 5’-terminal region of the *PTENP1 *pseudogene. The *PN2 *region of the *PTEN *gene promoter (*Fig. 2*) was analyzed using primers developed by the authors. The methylation status of the *PN4 *and *PN5 *loci was analyzed using primers [38] and [32], respectively.

**Table 1:** PCR primers and conditions used to determine the methylation status of promoters of the PTEN gene and PTENP1 pseudogene

Primer	Nucleotide sequence, 5’->3’	T_a._, °C	Number of PCR cycles	PCR product size, b.p.
PNP1-U-F	TTGTAGTTGTGATGGAAGTTTGAAT	64	33	156
PNP1-U-R	CCACCCCCACAAATACTCACA			
PNP1-M-F	TGTAGTCGTGATGGAAGTTTGAAT	63	33	152
PNP1-M-R	CCCCCGCGAATACTCACG			
PN2-U-F	TTGTAGTTATGATGGAAGTTTGAG	61	33	165
PN2-U-R	CCACCACCACAAACCAACCA			
PN2-M-F	TTGTAGTTATGATGGAAGTTTGAG	61	33	162
PN2-M-R	CGCCGCAAACCGACCGA			
PN4-U-F	GTTGGGGTGTGTGGAGTTTGGTT	61	36	135
PN4-U-R	CCCTCAAACTCCAAATCAATTCACAA			
PN4-M-F	CGCGCGGAGTTTGGTTTCG	62	32	117
PN4-M-R	CAAATCGATTCGCGACGTCG			
PN5-U-F	TATTAGTTTGGGGATTTTTTTTTTGT	60	36	186
PN5-U-R	CCCAACCCTTCCTACACCACA			
PN5-M-F	GTTTGGGGATTTTTTTTTCGC	60	36	178
PN5-M-R	AACCCTTCCTACGCCGCG			

Note. M – methylated; U – unmethylated; F – a forward primer; R – a reverse primer; PNP – primers for amplification of pseudogene regions; PN – primers for amplification of PTEN gene regions.

**Statistical analysis**

The statistical analysis was performed using the SPSS v.22 software package (SPSS Inc.). Differences in the methylation rates of the *PTENP1 *pseudogene among groups were assessed using a two-sided Chi-square test and the Fisher exact test. The statistical differences in the mean age among groups were assessed using the Mann-Whitney test.

**Table 2:** Methylation of promoters of the PTEN gene and PTENP1 pseudogene in normal, hyperplastic, and malignant endometrial tissues

Gene	Blood	Normal endometrium, mean age (extreme values)		Hyperplasia without atypia	Hyperplasia with atypia	Endometrial carcinoma
		24 (17–34)	52.5 (45–65)			
PTEN	0.00 (0/25)*	0.00 (0/24)	0.00 (0/19)	0.00 (0/30)	0.00 (0/13)	0.00 (0/57)
PTENP1	0.00 (0/25)^*^	4.17 (1/24)	57.89 (11/19)	73.33 (22/30)	76.92 (10/13)	70.83 (17/24)^**^
P		< 0.001^***^		< 0.351^****^	< 0.450^****^	< 0.521^****^

Note. Two-sided Fisher exact test.

^*^First digit – a percentage of methylated DNA samples; second digit – the number of methylated samples; third digit – the total number of samples.

^**^A subgroup of patients with age similar to that of the reference control group was selected from the main group of 57 patients with EC.

^***^Normal endometrium 24 (17–34) vs. 52.5 (45–65).

^****^Compared to normal endometrium 52.5 (45–65).

## RESULTS

In this study, we examined three regions near the minimal promoter of the *PTEN *gene, including CpG sequences, which could have potentially been methylated and not analyzed previously in EC (*Fig. 2*). A sequence flanked by *PN2 *primers is located 685 bp upstream of the ATG-codon and adjoins directly to the minimal promoter. The region situated between *PN4 *primers is located 1,913 bp upstream of the ATG codon. Methylation of this region was studied in melanoma [38]. A sequence situated between *PN5 *primers the methylation of which has already been studied in lung cancer is located 2,300 bp upstream of the ATG codon [32].

Starting the study, we first found that the *PTEN* gene was not methylated in the studied promoter regions in any of the DNA samples isolated from the analyzed tissues, including EC, EH, and normal endometrium (*Table 2*). Although this did not exclude genetic mutations in the gene, it indicated that the gene in our patients could not be inactivated by this epigenetic mechanism. Therefore, given the published data, we supposed that *PTEN *inactivation might occur via the known mechanism of ceRNA through suppression of *PTENP1* pseudogene transcription by methylation of its 5’-terminal region.

Indeed, a high rate of *PTENP1 *methylation was found in all endometrial tissue samples, except for the normal endometrium of young females (*Table 2*). At the same time, 73% (8 out of 11, *Table 2*) of normal endometrium samples from MAE females with methylated* PTENP1 *were mosaic: i.e., they contained some amount of cells with an unmethylated or partially methylated pseudogene (data not shown). Mosaic methylation of the pseudogene was also detected in several endometrial tissue samples of patients with EH and EC (e.g., *Fig. 1*, lines *3u *and *3m*), which might be due to contamination of tumor biopsy samples by normal cells. Methylation was tissue-specific and was absent in the blood of patients. Comparison of the *PTENP1 *methylation rates in the EC and EH groups and in the control group of MAE females did not reveal statistically significant differences between them (0.45 < *p * < 0.35). In all groups, the methylation level was high (71–77% in patients *vs*. 58% in control subgroup 2). At the same time, the mean age of the EC and EH patients included in the study was similar to the mean age of the females in control subgroup 2 (54.3, 52.1, and 52.5 years, respectively). There were also no significant differences in *PTENP1* methylation in subgroups of the main EC group, where patients were divided based on clinical and pathological characteristics: age, disease stage, depth of tumor invasion into the myometrium, differentiation degree of cancer cells, and tumor subtypes (*Table 3*). However, statistically significant differences in the rates of *PTENP1* methylation were found in the normal endometrium of young females (4%) and MAE females (58%) (*p * < 0.001). These results were unexpected. They suggest that *PTENP1* pseudogene methylation reflects primarily age-related changes in the human body and is not directly related to the studied endometrial pathology.

## DISCUSSION

In 2001, Salvesen and co-workers tried to analyze the methylation status of the *PTEN *gene promoter in EC. Methylation was detected in 19% of patients. However, these data proved to be erroneous because they did not take into account the high homology between the gene and its pseudogene *PTENP1 *[39]. Later, Zysman and co-workers also studied the methylation of the *PTEN* promoter region in EC [40]. In this case, *PTEN*-specific primers were used for methyl-sensitive PCR. Two sites were analyzed: the first one – in the minimal promoter of the *PTEN *gene; the second – near the ATG codon. Both sites were found to be unmethylated. These data suggest that studies of the *PTEN *gene promoter using primers not differentiating between *PTEN *and its pseudogene have detected methylation of *PTENP1*, but not *PTEN*. The methylation status of other *PTEN *gene regions in EC and EH has no longer been analyzed using *PTEN*-specific primers. However, methylation of other promoter regions of this gene was also found in other oncological diseases [32, 38, 41]. Therefore, we supposed that EC tissues might contain methylated sequences of the *PTEN* gene promoter region, with some of them having been previously analyzed and some being new, not studied yet in this disease. Thus, we decided to study a new locus located in the immediate vicinity of the minimum promoter, as well as two distally located sequences that had been analyzed previously (*Fig. 2*, see the Results section for more details). One of them, located between *PN4* primers, was methylated in 60% of melanoma cases [38].

We demonstrated that the analyzed sequences of the *PTEN *gene promoter region were not methylated in any of the studied tissue samples. Given these and published data, we concluded that methylation of the* PTEN *gene promoter was not involved in its inactivation in EC and EH, at least in our patients. Therefore, we supposed that suppression of *PTEN *gene expression in this case might occur via a competing endogenous RNA mechanism or via the involvement of asRNAs through inhibition of *PTENP1 *pseudogene transcription by methylation of its promoter region.

In fact, we found a high rate of methylation of the 5’-terminal *PTENP1 *region in patients with EC (70.83%), as well as EH with and without atypia (76.92% and 73.33%, respectively, *Table 2*). These results are consistent with the published data. In particular, methylation of the 5’-terminal region of the *PTENP1 *pseudogene was detected in 66% of small cell lung cancer cases [32]. However, pseudogene methylation in the endometrial samples of healthy females aged 45–65 years was unexpected. Methylation in this control subgroup occurred in 57.89% of cases. At the same time, *PTENP1* methylation occurred in only 4% of healthy young females aged 17 to 34 years (*Table 2*). The differences in the DNA methylation rates in these subgroups were statistically significant (*p* < 0.001). A further analysis revealed no significant differences in the rates of* PTENP1 *methylation between healthy MAE females and patients with EC and EH. Also, there was no correlation between pseudogene methylation and EC stages, cell differentiation degree, depth of invasion into the myometrium, or cancer subtypes (*Table 3*). It should also be emphasized that there is an absence of methylation in the blood of EC patients with pseudogene methylation in the endometrium. These facts indicate the tissue-specific nature of this phenomenon that accompanies changes in a healthy endometrium during aging of the human body.

**Table 3:** Association of PTENP1 pseudogene methylation with the clinical and pathological characteristics of endometrial cancer patients

Clinical and pathological characteristics	n	M(U)	M, %	p
Mean age (extreme values)				0.784
55 (48–60)	27	19(8)	70.37	
68 (61–76)	30	20(10)	66.66	
FIGO stage				1.00
I	46	31(15)	67.39	
II + III	11	8(3)	72.73	
Invasion into endometrium				0.359
Yes	17	10(7)	58.82	
No	40	29(11)	72.50	
Tumor differentiation				0.774
high (G1)	31	21(10)	67.74	
moderate and low (G2 + G3)	26	19(7)	73.08	
Bokhman subtype				
I	19	14(5)	73.68	1.00
II	11	8(3)	72.73	

Note. Two-sided Fisher exact test; M – methylated; U – unmethylated.

At present, we do not know how the discovered methylation of the studied *PTENP1 *pseudogene region affects its expression and the expression of the *PTEN* gene. There are three potential consequences of the methylation: no effect on *PTEN *expression; suppression of *PTEN *activity; and stimulation of *PTEN *activity. The three *PTENP1*-lncRNAs (*Fig. 2*) synthesized from the studied promoter, which were mentioned in the Introduction section, have an opposite effect [27]. *PTENP1*- asRNA-α inhibits *PTEN *transcription through heterochromatinization of its promoter by trimethylation of histone H3 in this chromatin region. *PTENP1*-sRNA that competes with *PTEN*-mRNA for miRNA acts as ceRNA, and *PTENP1*-asRNA-β stabilizes *PTENP1*- sRNA. The physiological outcome of pseudogene methylation will depend on changes in the ratio among these three *PTENP1*-RNAs in endometrial cells. Stabilization or stimulation of *PTEN *tumor suppressor gene activity during preferential simultaneous synthesis of *PTENP1*- sRNA and *PTENP1*-asRNA-β can protect against carcinogenesis. The consequence of suppressing its activity may be dual. Partial *PTEN *inactivation via this epigenetic mechanism may be a marker of the precancerous state of endometrial cells. At the same time, its complete rapid inactivation might also perform protective functions in aging endometrial cells.

Recently, the P. Pandolfi group discovered a new PTEN-dependent mechanism of cell aging, which was called *PTEN*-loss-induced cellular senescence (PICS) [42]. Unlike classical aging mechanisms, e.g., due to hyperactivation of oncogenes, PICS (at least in mice [42] and the primary cells of human epithelium [43]) can rapidly develop in nonproliferating cells in the absence of a cellular response to DNA damage. In this case, the development of PICS depends on the activity level of intracellular PTEN. Cell aging and cell cycle blockage via this mechanism in nonmalignant cells occur upon complete inactivation of *PTEN*, whereas its partial inactivation may be accompanied by the initiation of carcinogenesis and proliferation of malignized cells [42]. Therefore, we may suppose that *PTEN *that is partially inactivated by somatic mutations in endometrial cells bears the risk of cell malignant transformation. Therefore, complete suppression of the cell cycle and prevention of tumor growth via this mechanism require rapid complete inactivation of *PTEN*. This could apparently occur via the suppression of *PTENP1 *pseudogene transcription through methylation of its promoter and/or depletion of ceRNA, whose function is performed by* PTENP1*-sRNA. If this assumption is correct, *PTENP1* methylation may be considered as one of the elements of protection from aging cells with a high risk of malignant transformation. In this case, the *PTENP1 *methylation found in EH and EC cells may be a consequence of a preceding or still ongoing fight with their malignant transformation. To confirm or disprove this model, further studies of the effect of *PTENP1 *methylation on the expression of the *PTEN *gene are required.

## CONCLUSION

This study of normal tissues, malignant tumors, and endometrial hyperplasias in females of different ages revealed that the promoter region of the *PTEN *tumor suppressor gene was not methylated in any of the cases. In contrast to this, the bi-directional promoter of the* PTENP1 *pseudogene was methylated at a high frequency in all studied tissues, except for the endometrium of young healthy females, as well as the blood of endometrial cancer patients. We think that *PTENP1* pseudogene methylation reflects the age-related changes in the human body and may not be directly related to the studied endometrial pathology. We suggest that, depending on the effect of methylated *PTENP1* on the expression of the *PTEN *gene, pseudogene methylation may protect the body from the development of EC or serve as a marker of a precancerous state of cells. To select between these alternatives, it is necessary to further investigate the effect of *PTENP1 *methylation on *PTEN *gene expression in cultured human cells.

## Abbreviations

asRNA: antisence RNA
ceRNA: competing endogenous RNA
EC: endometrial carcinoma
EH: endometrial hyperplasia
lncRNA: long non-coding RNA
MAE: middle-aged (45–55 years) and elderly (> 55 years) females
miRNA: micro RNA
MRE: microRNA recognition element
PICS: PTEN-loss-induced cellular senescence
PTEN: phosphatase and tensin homolog
sRNA: sense RNA
